# Physiological responses of hydroponically-grown Japanese mint under nutrient deficiency

**DOI:** 10.7717/peerj.7751

**Published:** 2019-09-26

**Authors:** Chananchida Janpen, Naruemon Kanthawang, Chaiartid Inkham, Fui Ying Tsan, Sarana Rose Sommano

**Affiliations:** 1 Department of Plant and Soil Science, Faculty of Agriculture, Chiang Mai University, Chiang Mai University, Chiang Mai, Thailand; 2 Plant Bioactive Compound Laboratory (BAC), Faculty of Agriculture, Chiang Mai University, Chiang Mai University, Chiang Mai, Thailand; 3 Science and Technology Research Institute, Chiang Mai University, Chiang Mai University, Chiang Mai, Thailand; 4 Faculty of Plantation & Agrotechnology, Universiti Teknologi Mara, Universiti Teknologi Mara, Shah Alam, Malaysia

**Keywords:** Abiotic stress, Antioxidative mechanism, *Mentha arvensis*, Nutrient stress, Visual symptoms

## Abstract

This research aims to determine growth and deficiency patterns as well as antioxidative potentials of Japanese mint (*Mentha arvensis*) hydroponically grown under limited macronutrients and micronutrients. The experiment was conducted for 60 days after transplanting in an evaporative greenhouse (avg temp = 28–30 °C, 60–65 %RH), using deep water culture technique. Plants were grown in nutrient solution consisting of complete Hoagland’s solution (CTRL), and nutrient solutions lacking one of the following macronutrients and micronutrients: nitrogen (-N), phosphorus (-P), potassium (-K), iron (-Fe), manganese (-Mn), and copper (-Cu). The deficiency symptoms, growth patterns, and stress response mechanism were followed. All treatments except for the CTRL induced deficiency symptoms and physiological changes. Macronutrient deprivation reduced growth determined by the morphological parameters while micronutrient omission had no effect except for no iron treatment. The result showed that potassium and iron deficiencies had foremost adversely effect on growth of Japanese mint. Under nutrient stress conditions, plant only gave antioxidative responses to phosphorus and potassium deficiencies. However, the negative plant-stress relationship was found for no iron treatment indicating the detoxification mode of plant for lacking of micronutrient.

## Introduction

Japanese mint (*Mentha arvensis*) is an essential oil-bearing herbaceous plant belonging to the Lamiaceae family. Arial parts (viz., leaves, young stems, and parts of the inflorescence) comprise of glandular trichomes containing essential oil known particularly as mint oil with high market needs in cosmetics, pharmaceutical, food and flavoring industries (Gershenzon, McConkey & Croteau, 2000). The current world’s production of this mint oil reached nearly 48,000 tons annually (Sally, 2017). Much concern has been expressed lately on soil deterioration and unfavorable weather conditions due to the climate changes. These factors, as a consequence, largely affected production volume and quality of Japanese mint (Khaledian et al., 2017; Taneja & Chandra, 2012). To overcome such problems, urban farmers are beginning to explore alternative forms to grow plants. Hydroponics is a plant production technique in which root system contact directly to the solution which all nutrient elements are in a readily available form for plants to absorb and utilize (Schwarz, 1995). This system provides lists of advantages including increasing yield, minimizing production time, and more importantly is sustainable for urban environment (Jones, 2016).

Macronutrients and micronutrients are essential for plant growth and development through their involvement in numerous physiological processes and being cofactors required by several enzymes of primary and secondary metabolism (Marschner, 2012). The absence of these elements can cause plant abnormal growth and antagonistic symptoms that are specific to individual nutrient elements and plant species. It can also induce changes in the biosynthesis of primary and secondary compounds (Verma & Shukla, 2015). Such the abiotic stress can also lead to the over production of Reactive Oxygen Species (ROS) that cause oxidatively toxic effect to lipids, proteins, and nucleic acids of plant cells. Through biological process with or without stress, plants produce ROS in various forms including hydrogen peroxide (H_2_O_2_), superoxide anion (O_2_^•−^) and singlet oxygen (^1^O_2_) which are produced in the organelles such as chloroplast, peroxisomes, and mitochondria (Gill & Tuteja, 2010). Plants nonetheless has the system to protect themselves against the ROS through antioxidant defensive systems and accumulation of enzymatic and non-enzymatic secondary compounds to detoxify the harmful effect of ROS (Apel & Hirt, 2004; Gill & Tuteja, 2010). The accumulation of these compounds is beneficial particular for those plants with medicinal and aromatic properties (Pradhan et al., 2017).

Environmental threats have led to a risen demand for urban agricultural practice to grow food with an increasing intention to high value crop such as medicinal plant like Japanese mint. To this end, there is an inadequate information regarding nutrient management, the deficiency symptoms, and antioxidative responses of hydroponically grown mint under nutrient deficiency conditions. As for providing such information, the objective of this study is to define the deficiency symptoms, the effects on growth and antioxidative defensive mechanism of Japanese mint.

## Materials and Methods

### Plant material

Eight-week old Japanese mint cuttings (8-cm high), which were maintained using propagation standard condition of Thai Royal Project protocol (80% humidity greenhouse and daily irrigation once a day), were purchased from Khun Pae Royal Project, Chom Thong, Chiang Mai, Thailand. The cuttings were grown in coconut dust for 2 months prior to transferring to deep water culture hydroponic system.

### Treatments and growth conditions

Hoagland’s standard nutrient solution was prepared in this experiment (Hoagland & Arnon, 1950) with the additional of 7 different nutrient solutions as followed; (i) a complete solution (CTRL) consisting of 210 mg N, 30 mg P, 234 mg K, 200 mg Ca, 64 mg S, 48 mg Mg, 0.5 mg B, 5 mg Fe, 0.5 mg Mn, 0.02 mg Cu, 0.05 mg Zn, and 0.01 mg Mo per L. The other six treatments were identical to the CTRL without (ii) N (-N), (iii) P (-P), (iv) K (-K), (v) Fe (-Fe), (vi) Mn (-Mn) and (vii) Cu (-Cu). The treatments were arranged in a Randomized Complete Block Design (RCBD) in three replicates. The cuttings were washed and then transferred to each treatment. A continuous oxygen supply was added via air pump and air stone. The pH of all treatments was maintained at 6.5–7.0 with electrical conductivity (EC) at 1,900–2,000 µS/cm. The experiment was conducted in the period of 60 days as suggested as commercial harvesting time for this particular crop (Taneja & Chandra, 2012). Plants were grown in an evaporative cooling system greenhouse at Mae-Hia Agricultural Research, Demonstrative and Training Centre, Faculty of Agriculture, Chiang Mai University. During the experiment, the temperature of the greenhouse ranged from 28 to 30 °C, with 30% penetration of light and relative humidity at 60–65%.

### Morphological parameters

Every 15 day-period, the visual symptoms of deficiency of Japanese mint plants grown in each treatment were observed. Plant height (cm) and total dry weight (g) were recorded. Leaf area (cm^2^) was measured using LI-3100 leaf area meter (LI-COR, USA). Root system and aerial part were separated and then oven dried at 40 °C for 48 h. Dry weight of each part was used to calculate the shoot-to-root ratio (Bessa et al., 2013; Ericsson, 1995).

### Plant stress mechanism

#### Estimation of chlorophyll

Non-destructive chlorophyll estimation was determined as SPAD value from the first pair of fully expanded leaves (young) and the last pair of leaves (old) by using a portable SPAD-502 Plus chlorophyll meter (Konica Minolta, Japan).

#### Determination of electrolyte leakage

Electrolyte leakage was determined as described by García-Mata & Lamattina (2001) with slight modification. Leaf samples (15-cm^2^ pieces) were submerged into ten mL deionized water and kept at room temperature over 2 h. After the incubation, the initial electrical conductivity of the bathing solution (ECi) was measured using Eutech CON 700 conductivity meter (Eutech Instruments Pte Ltd, Singapore). Then, the samples were heated at 80 °C for 2 h to release all electrolytes, and the electrical conductivity of the heated bathing solution (ECt) was measured again. The percentage of electrolyte leakage was calculated using the following equation;

\begin{eqnarray*}\text{Electrolyte leakage}(\text{%})=(ECi/ECt)\times 100. \end{eqnarray*}

#### Extraction of antioxidative compounds

Dried aerial part was powdered using a hand-held grinder (Philips, Netherlands) at speed level 1. Sample powder (0.05 g) was extracted with 500 µL of 95% (v/v) methanol twice. After centrifugation, the supernatants were combined and used for the analyses of total phenolic, total flavonoid content and radical scavenging activities.

#### Determination of total phenolic content

The total phenolic content was determined spectrophotometrically according to the Folin Ciocalteu colorimetric method (Kanatt, Chander & Sharma, 2007; Sommano, 2015) calibrating against the gallic acid standards. Briefly, 30 µL of the methanol extract was mixed with 60 µL of 10% (v/v) Folin-Ciocalteu reagent. Thereafter, within 8 min, (210 µL) NaHCO_3_ at 6% (w/v) was added. The solution was mixed and incubated in the dark at room temperature for 30 min. The absorbance was measured at 725 nm using SPECTROstar Nano Microplate Reader (BMG LABTECH, Ortenberg, Germany). Results were expressed as mg of gallic acid equivalent per g dry weight of sample (mg GAE/ g DW).

#### Determination of total flavonoid content

The total flavonoid content was determined using aluminium chloride colorimetric method (Wolfe, Wu & Liu, 2003). The combination of the methanol extract (25 µL), distilled water (125 µL), and 5% (w/v). NaNO_2_ (7.5 µL) were thoroughly mixed. After 5 min, 15 µL of AlCl_3_ was added and the mixture was incubated at room temperature for 6 min. After which, NaOH (50 µL) and distilled water (27.5 µL) were added and mixed. The absorbance of this solution was measured at 510 nm using the Microplate Reader and compared with a standard curve of catechin solutions. The flavonoid content was expressed as mg of catechin equivalent per g dry weight of sample (mg CE/g DW).

#### Determination of DPPH radical scavenging activity

The 2, 2-diphenyl-1-picrylhydrazyl (DPPH) radical scavenging activity was measured using the method of Molan, De & Meagher (2009). The methanol extract (25 µL) was reacted with 0.2 mM DPPH-methanol solution (250 µL). The mixture was incubated in the darkness at room temperature for 30 min. The absorbance was measured at 517 nm. The percentage of the DPPH radical scavenging activity was calculated using the following equation;

\begin{eqnarray*}\text{DPPH radical scavenging activity}(\text{%})=[({A}_{\mathrm{control}}-{A}_{\mathrm{sample}})/{A}_{\mathrm{control}}]\times 100 \end{eqnarray*}

where; A_control_ is the absorbance of control [using 95%(v/v) methanol instead of sample].

A_sample_ is the absorbance of mixture of sample extract and 0.2 mM DPPH-methanol solution.

#### Determination of ABTS radical scavenging activity

The 2, 2′-azino-bis-(3-ethylbenzothiazoline-6-sulfonate) (ABTS) radical cation scavenging activity was measured using the method of ABTS radical cation decolorization assay (Re et al., 1999). To prepare the 7 mM ABTS stock solution, ABTS was dissolved at a 7 mM concentration and mixed with 2.45 mM potassium persulfate solution in the ratio of 1:1 (v/v). The stock solution was kept in the darkness at room temperature for 16 hrs. The ABTS working solution was diluted with phosphate buffer solution (2.4 mM, pH 7.4) until an absorbance of 0.7 ± 0.02 at 734 nm was obtained. Ten µL of the methanol extract was reacted with 200 µL of the ABTS working solution. The mixed solution was incubated at room temperature in the darkness for 30 min. The absorbance readings were recorded at 734 nm. The percentage of the ABTS radical scavenging activity was calculated using the following equation;

ABTS radical scavenging activity (%) = [(A_control_ − A_sample_) / A_control_] ×100

where; A_control_ is the absorbance of ABTS working standard.

A_sample_ is the absorbance of mixture of sample extract and ABTS working solution.

### Statistical analysis

The experiment was set up in a randomized complete block design, with seven treatments and three replicates. Fifteen plants were used in each replicate of a single treatment. The extractions of phytochemicals and analyses were of three separate lots of samples. The data were subjected to Analysis of Variance (ANOVA) and comparison between the mean values of treatment were confirmed by Tukey’s Honest Significant Difference (HSD) test at the 95% level of significance. Pearson’s correlation coefficients were calculated for various variables.

## Results

### Effects of individual macronutrient deficiency on growth of Japanese mint

#### Nitrogen deficiency

**Figure 1:**
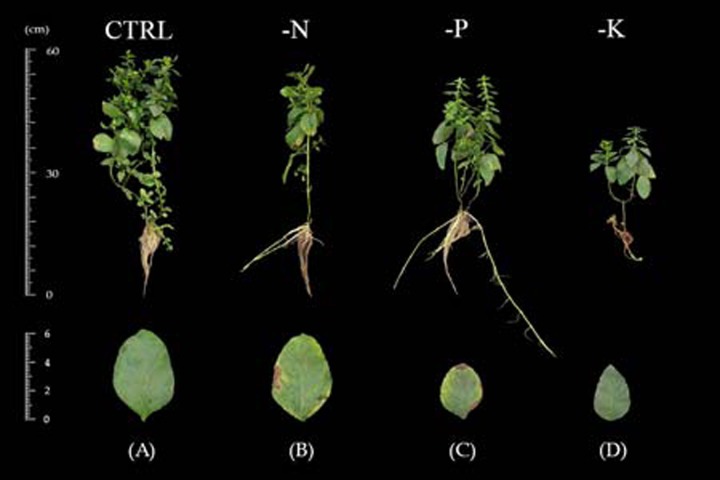
Growth and visual symptoms on old leaves of Japanese mint grown under macronutrient deficiency for 60 days after transplanting to the nutrient solution without individual macronutrient. (B) nitrogen (-N); (C) phosphorus (-P); (D) potassium (-K) in comparison with plants grown in (A) complete nutrient solution (CTRL).

**Figure 2:**
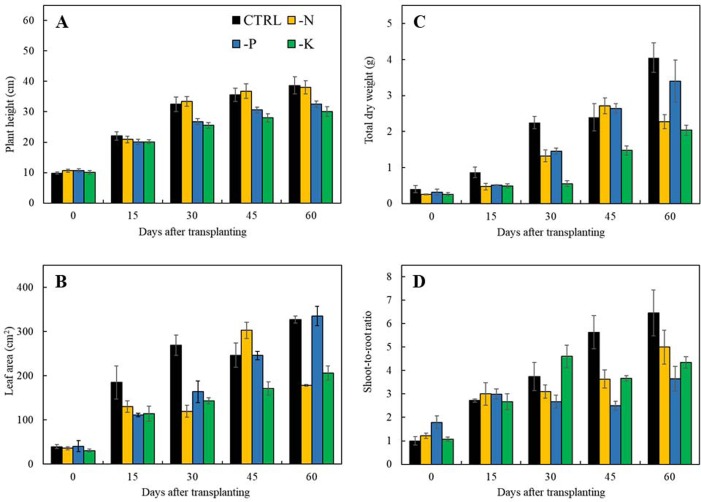
Morphological parameters of Japanese mint grown in nutrient solution with individual macronutrient deficiencies [(-N), (-P), and (-K)] after transplanting for 60 days. (A) plant height; (B) leaf area; (C) total dry weight; and (D) shoot-to-root ratio. Data are means of the replicate samples. Bars represent standard error.

The initial symptoms of nitrogen-deficient Japanese mint were observed at 15 days after transplanting. Plants grown in the -N treatment showed stunted growth and deficiency symptom of the yellowish green foliage. The symptoms appeared first in the old leaves which were chlorosis over the entire leaf and necrosis at the leaf margin (Fig. 1B). In term of plant productivity, nitrogen deficiency reduced leaf area, total dry weight and shoot-to-root ratio at 30 days after transplanting in comparison with plants grown in the CTRL (Figs. 2B–2D), while plant height was not affected (Fig. 2A).

#### Phosphorus deficiency

Phosphorus deficiency symptoms were clearly noticeable at 60 days of transplanting. The leaf blade became dark green with emerged purple color at the margin of the leaves. The old leaves showed chlorosis from the edge of the leaves with the progressing necrosis (Fig. 1C). Limits in phosphorus had negative effects on all growth parameters including plant height, leaf area, total dry weight, and shoot-to-root ratio after 30 days of transplanting (Figs. 2A–2D).

#### Potassium deficiency

Symptoms of potassium deficiency were apparent after 30 days of transplanting to the nutrient solution without potassium (-K). Plants showed stunted growth and became wilted with distributing yellow spots in old leaves blade during 60 days of experiment (Fig. 1D). Limits in potassium had also an adversely negative effect on plant height, leaf area, and total dry weight (Figs. 2A–2C) after 30 days of transplanting as compared to plants grown in the CTRL. Shoot-to-root ratio decreased at 45 days after transplanting (Fig. 2D).

### Effects of individual micronutrient deficiency on growth of Japanese mint

#### Iron deficiency

**Figure 3:**
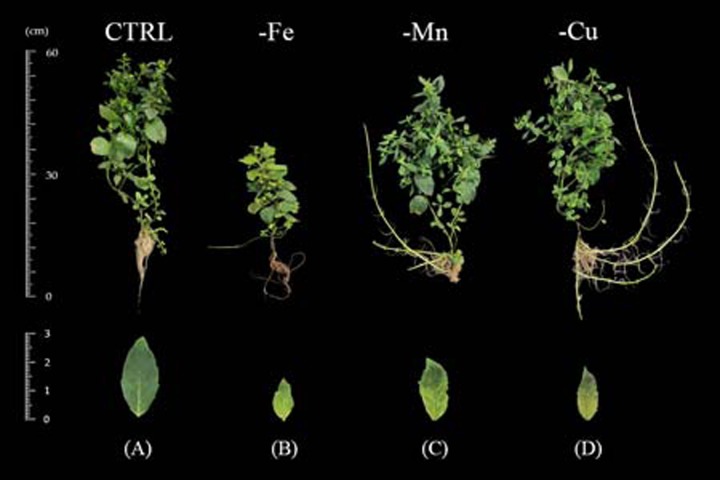
Growth and visual symptoms on young leaves of Japanese mint grown under micronutrient deficiency for 60 days after transplanting to the nutrient solution without individual micronutrient. (B) iron (-Fe); (C) manganese (-Mn); (D) copper (-Cu) in comparison with plants grown in (A) complete nutrient solution (CTRL).

**Figure 4:**
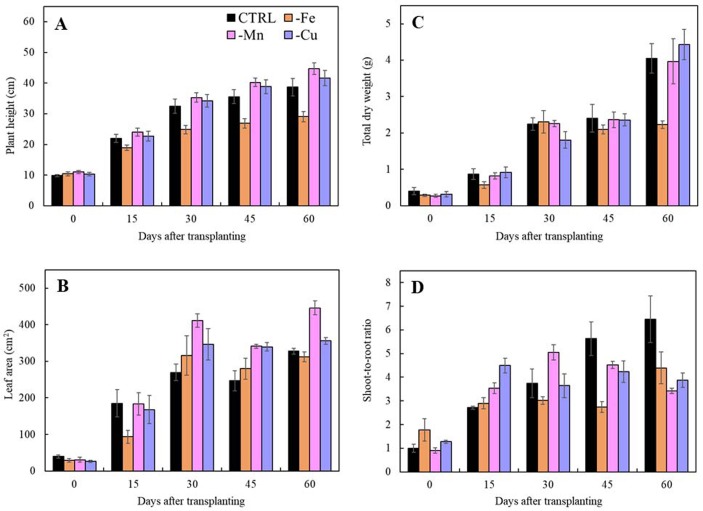
Morphological parameters of Japanese mint grown in nutrient solution with individual micronutrient deficiencies [(-Fe), (-Mn), and (-Cu)] after transplanting for 60 days. (A) plant height; (B) leaf area; (C) total dry weight; and (D) shoot-to-root ratio. Data are means of the replicate samples. Bars represent standard error.

Iron deficiency symptoms were noticeable at 7 days after transplanting to the -Fe treatment. Young leaves showed chlorosis which initiated at the base of the petiole and expanded toward the leaves (Fig. 3B). This symptom was progressive to the older leaves illustrating interveinal chlorosis until 60 days after transplanting. Limits in iron retarded plant height and leaf area after 15 days of transplanting (Figs. 4A–4B). In addition, shoot-to-root ratio of the iron-deficient plants decreased after 30 days and total dry weight decreased at 45 days after transplanting (Figs. 4C–4D).

#### Manganese deficiency

The visual symptoms of manganese deficiency occurred at 30 days after transplanting. The shoot apex showed distortion and necrosis (Fig. 3C). The young fully expanded leaves exhibited chlorosis from the base of petiole and progressing toward the middle of the leaves. Moreover, the oldest leaves had also chlorosis at the leaf margin. In term of the productivity, manganese deficiency had no effect on plant height, leaf area, and total dry weight (Figs. 4A–4C) whereas shoot-to-root ratio decreased after 45 days of transplanting (Fig. 4D) when compared with the CTRL. However, Manganese-deficient Japanese mint seemed to have normal growth.

#### Copper deficiency

Copper deficiency symptoms were shown at 45 days after transplanting. Shoot tip was pale grey in color with necrosis and young fully expanded leaves were chlorosis (Fig. 3D). Moreover, progressing chlorosis in old leaves initiated from the edge of the leaf. Limits in copper had no effect on plant height, leaf area and total dry weight (Figs. 4A–4C), but reduced shoot-to-root ratio after 45 days of transplanting (Fig. 4D).

### Defensive mechanism of macro- and micronutrient deficiency

**Figure 5:**
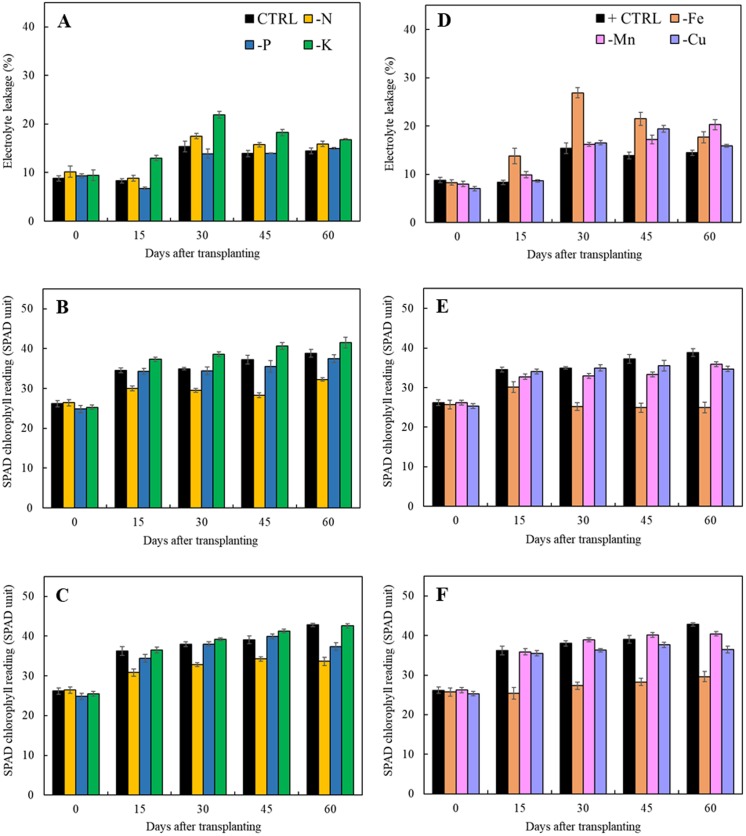
Electrolyte leakage as well as SPAD chlorophyll readings of young and old leaves of Japanese mint grown in solution with individual macro- and micronutrient deficiency after transplanting for 60 days. (A, D) Electrolyte leakage; (B, E) SPAD chlorophyll reading of young leaves; (C, F) SPAD chlorophyll reading of old leaves. Data are means of the replicate samples. Values are means ± S.E. Bars represent standard error.

Results in Fig. 5 indicated that lacking of both macro- and microelements increased electrolyte leakage from 15 days after transplanting except that of limits in phosphorus treatment as compared to the control. For the treatments with the omission of macronutrients, limitation of nitrogen and potassium had a negative impact on cell membrane integrity resulting in the increase of electrolyte leakage (Fig. 5A). Nitrogen deficiency also had adversely effect on the chlorophyll content as observed by reduction of SPAD value in both types of leaves (Figs. 5B and 5C). For the limits in micronutrient treatments, the electrolyte leakage increased after 45 days of transplanting (Fig. 5D). Iron and manganese deficiency had a negative impact on cell membrane stability resulting in the high increment of electrolyte leakage and reduction of SPAD values. (Figs. 5D and 5F).

The results present in Table 1 showed that the quantity of total phenolic, flavonoid, and antioxidant capacity of Japanese mints were influenced by lacking of macronutrients in comparison with those grown in the complete nutrient solution. Nitrogen and phosphorus deficiencies had no significant effects on total phenolic content while potassium deficiency gave the significantly highest amount of total phenolic (4.68 ± 0.39 mg GAE/g DW). Additionally, nitrogen and phosphorus deficiencies significantly increased total flavonoid content (4.37 ± 0.49 and 4.65 ± 0.25 mg CE/g DW). They had also significantly increased DPPH radical scavenging activity (37.53 ± 0.39% and 42.77 ± 0.88%) when compared with the CTRL (28.08 ±0.30%) while significantly reduced in phosphorus-deficient plants (23.91 ± 0.96%). However, omission of macronutrient deficiency had no significant effect on ABTS radical scavenging activity. Growing the Japanese mint under the hydroponic system with limits in micronutrients (Fe, Mn, and Cu) for 60 days after transplanting, had no effect on both total phenolic content and ABTS radical scavenging activity but significantly increased total flavonoid content (0.0051, 0.0062, and 0.0053 g CE/g DW, respectively). Moreover, DPPH radical scavenging activity was lower in the iron-deficient plants (20.34 ± 0.36%) and higher in copper-deficient plants (32.71 ± 0.95%) as compared to those of the CTRL. However, manganese deficiency had no effect on DPPH radical scavenging activity.

**Table 1:** Total phenolic, flavonoid contents and radical scavenging activities of Japanese mint grown under limit of an individual macro- and micronutrient at 60 days after transplanting.

**Treatments**	**Total Phenoliccontent(mg GAE/g DW)**	**Total flavonoid content(mg CE/g DW)**	**DPPH radical scavenging activity (%)**	**ABTS radical scavenging activity (%)**
CTRL	3.20 ± 0.21^a^	2.33 ± 0.29^a^	28.08 ± 0.30^c^	84.19 ± 1.31^ab^
Macronutrients				
-N	3.31 ± 0.01^a^	4.37 ± 0.49^bc^	37.53 ± 0.39^e^	88.79 ± 0.58^ab^
-P	2.77 ± 0.41^a^	4.65 ± 0.25^bc^	23.91 ± 0.96^ab^	81.72 ± 2.95^ab^
-K	4.68 ± 0.16^b^	3.75 ± 0.15^ab^	42.77 ± 0.88^f^	90.06 ± 0.21^b^
Micronutrients				
-Fe	2.40 ± 0.04^a^	5.09 ± 0.06^bc^	20.34 ± 0.36^a^	80.94 ± 0.68^a^
-Mn	3.09 ± 0.02^a^	6.19 ± 0.19^c^	27.16 ± 0.20^bc^	87.65 ± 0.20^ab^
-Cu	2.80 ± 0.21^a^	5.35 ± 0.26^bc^	32.71 ± 0.95^d^	85.92 ± 1.20^ab^

**Notes.**

Results are means ± S.E. of three replicates. Different uppercase letters in the same column indicate significant differences according to Tukey’s HSD test at the 0.05 level.

DW dry weight GAE Gallic acid equivalents CE Catechin equivalent DPPH 2, 2-diphenyl-1-picrylhydrazyl radical scavenging activity ABTS 2, 2′-azino-bis-(3-ethylbenzothiazoline-6-sulfonate) radical cation scavenging activity

Our results found the relationship between stress responses (electrolyte leakage and chlorophyll apparatus damage as determined by SPAD chlorophyll reading) and antioxidative mechanism [total phenolic and total flavonoid content and antioxidative potentials (via DPPH and ABTS assay)] as shown in Table 2. Among the macronutrient deficiency, phosphorus and potassium had negative correlation between SPAD chlorophyll reading and antioxidant activities which indicate antioxidative response mechanism to stress. Nonetheless, micronutrient deficiency revealed the opposite trend by illustrating negative correlation between electrolyte leakage and ABTS radical scavenging potential of the iron stress mint.

**Table 2:** Correlation between stress responses and antioxidative mechanism of Japanese mint grown under an individual macro- and micronutrient deficiency at 60 days after transplanting.

	**Stress responses**					
**Antioxidative responses**	**Electrolyte leakage**			**SPAD chlorophyll reading**		
	Macronutrients					
	- N	- P	- K	- N	- P	- K
TPC	*r* = − 0.334	*r* = − 0.327	*r* = − 0.876	*r* = 0.629	*r* = 0.854	*r* = − 0.401
TFC	*r* = − 0.613	*r* = − 0.191	*r* = − 0.315	*r* = 0.315	*r* = − 1.000^*^	*r* = − 0.894
DPPH	r = 0.645	*r* = 0.463	*r* = − 0.117	*r* = − 0.354	*r* = 0.904	*r* = − 1.000^*^
ABTS	r = 0.989	*r* = − 0.633	*r* = 0.491	*r* = − 0.885	*r* = 0.622	*r* = 0.823
	Micronutrients					
	- Fe	- Mn	- Cu	- Fe	- Mn	- Cu
TPC	*r* = − 0.969	*r* = − 0.828	*r* = 0.519	*r* = − 0.624	*r* = − 0.369	*r* = − 0.969
TFC	r = 0.673	*r* = − 0.934	*r* = 0.995	*r* = − 0.399	*r* = − 0.570	*r* = − 0.641
DPPH	*r* = − 0.993	*r* = 0.991	*r* = 0.877	*r* = − 0.299	*r* = 0.743	*r* = − 0.963
ABTS	*r* = − 0.999*	*r* = − 0.674	*r* = 0.098	*r* = 0.446	*r* = − 0.973	*r* = − 0.767

**Notes.**

r, correlation coefficient

* significant correlation at the 0.05 level.

TPC total phenolic content TFC total flavonoid content DPPH 2, 2-diphenyl-1-picrylhydrazyl radical scavenging activity ABTS 2, 2′-azino-bis-(3-ethylbenzothiazoline-6-sulfonate) radical cation scavenging activity

## Discussions

### Effects of individual macronutrient deficiency on growth of Japanese mint

Nitrogen deficiency generally effected plant growth with chlorosis symptoms initially in old leaves toward young leaves. Marschner (2012) described that the incident was due to high mobility of the nutrient through phloem. These results were as expected since nitrogen plays an important role in plant growth with regard to the synthesis of major molecules including proteins, nucleic acids, hormones, and chlorophyll (Hopkins & Hüner, 2004). Additionally, nitrogen atoms are also parts of the porphyrin ring in the chlorophyll molecule which chelated to magnesium ion. Limiting N is therefore symptomized by lacking of the greenness basically from the incomplete structure of chlorophylls (Barker & Pilbeam, 2007). Our results are in line with Boussadia et al. (2010) and Silva et al. (2014), the largest reduction in plant growth based on dry matter was also observed in nitrogen-deficient olive and young peppermint (*Mentha piperita*) as compared with the complete nutrient solution. Hörtensteiner & Feller (2002) described that on-going nitrogen starvation caused the breakdown of leaf nucleic acids and proteins which are usually associated with leaf senescence.

For phosphorus deficiency, similar symptoms as shown in our results were also apparent in sweet basil and lavender in which the limits in phosphorus adversely affected dry matter yield of the leaves when compared with plant hydroponically grown in the complete nutrient solution (Borges et al., 2016; Chrysargyris, Panayiotou & Tzortzakis, 2016). In this same research, the visual symptoms were also characterized by darker green in old leaves and distributed to younger leaves because the higher nutrient mobility. Taiz & Zeiger (2002) described this incident of intense green coloration was associated with the accumulation of other pigments. The decrease in shoot-to-root ratio was also observed in soybean and silver birch (*Betula pendular*) seedlings under limits in phosphorus conditions (Ericsson, 1995; Fredeen, Rao & Terry, 1989). The researchers urged that lower production yield (shoot-to-root ratio) was due to the increase in partitioning of carbohydrates towards the roots, indicated by a strong increase particularly in sucrose concentration of the roots. Moreover, Mollier & Pellerin (1999) explained that phosphorus deficiency in maize disrupted shoot growth leading to a lower demand for carbohydrates, thus more carbohydrates are available for root sinks.

The reduction in growth of Japanese mint and the deficiency symptoms of potassium deficiency were similar to the observation in yarrow (*Achillea millefolium* L.) which limited potassium restricted plant growth by decreased total dry matter. Additionally, this plant illustrated slight vigor and provided chlorosis at the edges of old leaves followed by freckle appearance in the central part of the leaves and eventually turned necrosis (Alvarenga et al., 2015). Thus, potassium has the primary role in the regulation of the osmotic potential in the cells which is evident in plant physiological processes such as pressure driven solute transport in the xylem and phloem and the opening and closure of stomatal guard cells. The amount of potassium ion induces the changes in guard cell volume and osmotic pressure which are associated with stomatal opening (Humble & Raschke, 1971; Maathuis, 2009). However, this trend is not usual. In other study, potassium deficiency prevents the transport of sugars into roots that normally leading to increase the shoot-to-root ratio (Cakmak, Hengeler & Marschner, 1994; Ericsson, 1995; Gerardeaux et al., 2009).

### Effects of individual micronutrient deficiency on growth of Japanese mint

Our finding in iron deprivation treatment is in agreement with Wasli et al. (2018) who noticed a depressive effect on the shoot height, dry weight and relative growth rate of iron-deficient dill (*Anethum graveolens* L.) grown in hydroponic system as compared with those grown in the complete nutrient solution. Marschner (2012) described that interveinal chlorosis of young leaves is the most obvious visible symptom of iron deficiency. The deficiency symptoms appeared first in young leaves may be due to the formation of insoluble compounds of iron with lower mobility. Iron is required for the structural and functional integrity of the thylakoid membranes (Platt-Aloia, Thomson & Terry, 1983), as well as for ferredoxin and the biosynthesis of chlorophyll. Lacking of Fe was also explained by sensitivity of chloroplast and the thylakoid in which resulted in a severe chlorosis in plants (Barker & Pilbeam, 2007).

Manganese is an essential micronutrient with a function as a catalyst in the oxygen-evolving complex of photosystem II in photosynthesis (Schmidt, Jensen & Husted, 2016). Manganese-deficient plants showed interveinal chlorosis, reflecting photosynthesis disturbance, which is only visible when plant is severely stressed. From our result, the Japanese mint seemed to have normal growth which in line with the results of Neves, De Sá & De Carvalho (2004) who also evaluated manganese deficiency in Umbuzeiro (*Spondias tuberosa* L.) seedlings.

The result for copper deficiency was in contrast with the research finding in copper-deficient yarrow, total dry matter was not affected while total chlorophyll content was reduced (Alvarenga et al., 2015). According to Taiz & Zeiger (2002) copper is part of enzymes such as plastocyanin which is involved in electron transfer during the light reactions of photosynthesis. The distortion of young leaves, chlorosis, and necrosis starting at the apical meristem extending to the leaf margins, a bleaching of young leaves are typical visual symptoms of copper deficiency (Sommer, 1945).

### Defensive mechanism of macro- and micronutrient deficiency

Plants required a strict balance of uptake, utilization, and storage of mineral elements for proper ion homeostasis. Biological responsive activities in plants occur at cellular membranes during unfavorable environments i.e., drought stress, salt stress, and nutrient stress (Shabala, 2017). These stresses induce ROS leading to oxidative damage of biomolecules (lipids, proteins and DNA), loss of cell membrane integrity and activate programmed cell death (Apel & Hirt, 2004; Das & Roychoudhury, 2014). Moreover, light reaction in the photosynthesis, which comprised of both energy transfer and electron transport, also accompanies by formation of ROS. Photosystem II act as a highly oxidizing reaction center when absorbs light energy that plants access photolysis to gain electron back from water molecules, thereby delivering oxygen as a by-product (Cakmak & Kirkby, 2008; Noctor, Reichheld & Foyer, 2018). Under normal circumstance, plant establishes scavenging system to detoxify these ROS (Foyer & Shigeoka, 2011). However, during severe stress condition, normal scavenging system is insufficient to eliminate excess ROS formation which may cause oxidative damage to polyunsaturated fatty acids in the chloroplast envelope and photosynthetic protein complex (Gill & Tuteja, 2010; Khanna-Chopra, 2012). Therefore, the extend of stress can be either justified by the degree of membrane stability which can be assessed by the electrolyte leakage from cells (Blokhina, Virolainen & Fagerstedt, 2003; Chaturvedi, Singh & Bahadur, 2012; Levitt, 1980) or damage to chlorophyll apparatus as described by reduction of total chlorophyll content (Pospíšil, 2016; Swapna & Shylaraj, 2017).

SPAD chlorophyll reading was suggested to be a rapid assessment of chlorophyll content as well as stress indicator throughout plant growth (Azia & Stewart, 2001; Minolta, 1989; Netto et al., 2005). SPAD readings <25 indicated the diminishment of leaf photosynthetic in tree plants Sycamore maple (*Acer pseudoplatanus*), oak tree (*Quercus robur*), and beech tree (*Fagus sylvatica*) (Percival, Keary & Noviss, 2008). However, *Netto et al. (2005)* advised that SPAD values <40 were correlated with reduction of photosynthetic efficiency in coffee leaves. Roosta, Estaji & Niknam (2018) advised that iron deprivation was more destructive effect to photosynthesis apparatus than that of zinc and manganese in lettuce of different cultivars. Excessive electrolyte leakage also suggested membrane damage and programmed cell death in iron-deficient *Arabidopsis* leaves (Msilini et al., 2009).

Many forms of environmental stress have been shown to increase levels of ROS in plant cells. In order to protect cells against the toxic effect of ROS, plants have complex antioxidant mechanisms that involve enzymatically and non-enzymatically antioxidant compounds (Mittler, 2002). Phenylpropanoids and flavonoids play important roles in plants which associated with multiple stress-related functions including scavenging of reactive ROS (Dixon & Paiva, 1995; Suzuki et al., 2012). Phenolic compounds are accumulated through the phenylpropanoid metabolism by phenylalanine ammonialyase (PAL), the key enzyme induced by various abiotic stresses (Solecka, 1997). To determine the antioxidant activity, the methods of ABTS ^•+^ and DPPH ^•^ scavenging assay are the most commonly used. Both are characterized by excellent reproducibility under certain assay conditions (Wojdyło, Oszmiański & Czemerys, 2007). Macronutrient-deficient Japanese mint had no effect on antioxidative mechanism detected by ABTS radical scavenging assay. Similar results were observed by Nguyen, Kwee & Niemeyer (2010) in basil grown with limits in potassium treatment (1.0 mM K level) in sand culture.

Micronutrients play the important role as metabolic enzyme cofactors. These cofactors are redox-active that are the basis for their occurrence as catalytically active cofactors in many metalloenzymes (Hänsch & Mendel, 2009). In another study, iron is required by many antioxidant enzymes as it is an important catalyst of electron transfer reactions (Bould et al., 1953; Wasli et al., 2018). Negative correlation between stress response and antioxidative mechanism was found for iron-deficient Japanese mint. For this explanation we estimated that at commercial harvesting time (60 days), the stressed plant may undergo detoxification or starvation recovery (Foyer & Shigeoka, 2011; Pan, Wu & Xie, 2019).

## Conclusions

The present study illustrated that macro- and micronutrient deficiencies in the hydroponically grown Japanese mint were manifested as visual deficiency symptoms. The most limiting macro- and micronutrient elements are potassium and iron which exhibited the adversely reduction of plant growth patterns when compared with plants grown under the complete nutrient solution. Omission of P, K and Fe may also induce the oxidative stress condition which disrupts cell membrane stability and chlorophyll apparatus. The accumulation of non-enzymatic antioxidant compounds, phenolics and flavonoids, was enhanced along with the increase in DPPH radical scavenging activity. These showed the defensive mechanism of plant under nutrient deficiency conditions. The findings reported herein will be valuable to support optimal cultivation Japanese mint cultured in the hydroponic system. Future research study will focus on those particular macro- and micronutrients with the terpenoid (menthol) biosynthesis of highly commercial demand.

## Additional Information and Declarations

### Data Availability

The following information was supplied regarding data availability: The measurement data is available at figshare: Janpen, Chananchida (2019): Physiological responses of hydroponically-grown Japanese Mint under nutrient deficiency. figshare. Dataset. https://doi.org/10.6084/m9.figshare.9199913.v1.
